# Elderly dialysis patients: analysis of factors affecting long-term survival in 4-year prospective observation

**DOI:** 10.1007/s11255-012-0166-4

**Published:** 2012-04-15

**Authors:** Katarzyna Madziarska, Waclaw Weyde, Magdalena Krajewska, Ewa Zukowska Szczechowska, Katarzyna Gosek, Jozef Penar, Renata Klak, Tomasz Golebiowski, Cyprian Kozyra, Marian Klinger

**Affiliations:** 1 Department of Nephrology and Transplantation Medicine, Wroclaw Medical University, Borowska 213 St., 50-556 Wroclaw, Poland; 2 Internal Medicine, Diabetology and Nephrology, Medical University of Silesia, Zabrze, Poland; 3 Department of Statistics, Wroclaw University of Economics, Wroclaw, Poland

**Keywords:** Elderly, Hemodialysis, Peritoneal dialysis, Survival, Extracellular volume control

## Abstract

**Purpose:**

To assess factors influencing the long-term survival of elderly dialysis patients.

**Methods:**

The study group consisted of 51 prevalent dialysis patients aged over 70 years (32 F and 19 M, all caucasians), who had been on a chronic hemodialysis (27) or peritoneal dialysis program (24) for at least 2 months; median age was 77 years, median time on dialysis before inclusion was 16 months, and median residual diuresis was 600 ml. The patients were prospectively followed up to 4 years, and an analysis of factors affecting survival was performed.

**Results:**

Thirteen patients from the initial cohort of 51 (25.5 %) survived the whole 48-month observation period: 10 HD patients (37 %) and 3 PD patients (12.5 %). Annual mortality rate was 28.2 %: 37.4 % on PD vs. 20.9 % on HD. The dialysis modality had a significant impact on patients’ survival (*p* = 0.049; Cox F-test). The independent mortality risk factors in the Cox proportional hazard regression model were higher plasma pro-atrial natriuretic peptide (pro-ANP) (*p* = 0.006), lower residual diuresis (*p* = 0.048), and lower systolic blood pressure (BP) value (*p* = 0.039).

**Conclusions:**

Paramount for the survival of the elderly on dialysis is adequate extracellular volume control. Residual renal function is a protective factor for the survival of elderly HD patients. This observation is novel, not previously reported in an elderly dialysis population.

## Introduction

The population of patients with chronic kidney disease is growing, despite progress in preventive measures, at a rate of 5–8 % per year [1, 2].

The greatest increase in the incidence and prevalence of end-stage renal disease (ESRD) is occurring among the elderly, who simultaneously constitute the most fragile subset of dialysis patients, with annual mortality reaching 25 % [3–6].

This is exemplified by data from the European registry showing that 48 % of new dialysis patients are above the age of 65 and have a 2-year survival rate of 51 % [7, 8].

A Canadian study found that patients older than 75 had survival of 20.3 % at 5 years after dialysis initiation [9]. Therefore, an important task of dialysis care is to reduce the excessive mortality of this frail geriatric cohort. A substantial step in this direction is the identification of potentially reversible factors negatively affecting the long-term survival of dialysis seniors.

This was the aim of our study encompassing a group of 51 elderly above 70 years, median age 77 years, who had been on maintenance dialysis at least 2 months, median period 16 months. The included patients were prospectively followed for 4 years. Additionally, in the study, we compared in these elderly patients the efficacy of hemodialysis (HD) and peritoneal dialysis (PD), because both dialysis modalities were nearly equally distributed.

## Materials and methods

The study group was created from 51 prevalent dialysis patients with aged over 70 years (32 F and 19 M, all Caucasian) who had been on a chronic hemodialysis (27) or peritoneal dialysis program (24) for at least 2 months; median age 77 years, median time on dialysis before inclusion 16 months, and median residual diuresis 600 ml.

The patients were recruited from three dialysis centers in southwest Poland (Wroclaw, Zabrze, Walbrzych).

Twenty-six patients (51 %) were diabetics with the highly advanced Monckeberg type of arteriosclerosis with medial intravascular calcifications in the forearm arteries demonstrated by X-ray.

The group was formed in 2006 and prospectively observed during the subsequent 4 years.

The patients included in the study were free of active infection, symptomatic coronary disease, overt heart failure, history of malignancy, and diseases requiring immunosuppressive treatment. The protocol was approved by the local ethics committee. Clinical data of patients were extracted from the hospital records.

The impact of the following factors on survival was tested: baseline characteristics (age, gender, and race—all caucasian), residual diuresis, duration of dialysis, all standard indicators of dialysis care (blood pressure, HGB, BMI, adequacy of dialysis—Kt/V), and the following laboratory parameters: serum pro-atrial natriuretic peptide (pro-ANP), serum N-terminal pro-B-type natriuretic peptide (NT-proBNP), serum C-reactive protein (CRP), interleukin 6 (IL-6), serum albumin, and cholesterol.

All HD patients were dialyzed using a native arteriovenous fistula fulfilling a single-pool Kt/V ≥ 1.3. All PD patients were treated by continuous ambulatory peritoneal dialysis (CAPD) achieving weekly Kt/V > 1.7. Kt/V was calculated from three consecutive measurements at monthly intervals.

Routine laboratory tests (HGB[g/dl], CRP [mg/l], serum albumin [g/dl], and cholesterol [mmol/l]) were measured in the Central Hospital Laboratory as part of the standard care.

In addition, serum pro-ANP (amino terminal 1–98 ANP fragment) and serum NT-proBNP were assessed by ELISA (BIOMEDICA, Vienna, Austria); interleukin-6 (IL-6) was measured by ELISA (R & D Systems, Minneapolis, USA).

The blood samples were taken in HD patients before the midweek dialysis session and in PD patients during a control visit at the outpatient clinic during morning hours before the first fluid exchange.

The statistical analysis was performed with Statistica 9.0 software. Univariate methods employed for the analysis were Pearson’s χ^2^ test of independence and Fisher’s exact test (testing associations between two categorical variables), and nonparametric Mann–Whitney U test (comparing means of qualitative variables in two groups). Multivariate analysis was conducted using the Cox proportional hazard model (investigating the influence of qualitative variables on risk of death), and tests of survival time equality in two groups (the Cox F-test and the log-rank test).

Statistical significance was recognized with a *p* value <0.05. For quantitative variables, results are given as mean ±SD.

## Results

Demographic, clinical, and laboratory parameters of the investigated patients at study entry are presented in Table 1. The data are shown separately for the HD and PD patients. There were two significant differences between the groups at the start of the study: significantly lower albumin level (*p* = 0.026) and significantly higher cholesterol concentration (*p* < 0.001) in PD compared to HD patients.

**Table 1:** Clinical characteristics of study groups

Characteristics	PD *n* = 24 pts (min-median-max)	HD *n* = 27 pts (min-median-max)	*n* = 51 pts (min-median-max)	Mann–Whitney U test *p* value
Age (years)	76.88 ± 3.62 (71-77-82)	78.00 ± 3.67 (71-78-86)	77.47 ± 3.65 (71-77-86)	0.320
Gender (M/F)	5/19 (20.8 %/79.2 %)	14/13 (51.9 %/48.9 %)	19/32 (27.3 %/62.7 %)	
BMI (kg/m2)	26.26 ± 3.97 (19.4-26.3-32.9)	26.22 ± 4.57 (16.8-26.1-37.8)	26.24 ± 4.26 (16.8-26.1-37.8)	0.917
Duration of dialysis (months)	18.79 ± 17.95 (2-15.5-80)	19.00 ± 13.42 (2-17-50)	18.90 ± 15.56 (2-16-80)	0.467
Systolic BP (mmHg)	127.42 ± 14.91 (100-122.5-158)	131.48 ± 14.92 (90-130-160)	129.57 ± 14.91 (90-130-160)	0.148
Diastolic BP (mmHg)	76.92 ± 9.09 (60-77.5-94)	79.63 ± 8.31 (60-80-95)	78.35 ± 8.71 (60-80-95)	0.232
Residual diuresis (ml/24 h)	642.50 ± 510.25 (0-650-1500)	564.81 ± 317.08 (0-500-1200)	601.37 ± 416.62 (0-600-1500)	0.691
Hb (g/dl)	11.33 ± 1.11 (9.4-11.25-13.9)	11.48 ± 1.29 (8.8-11.7-13.7)	11.41 ± 1.20 (8.8-11.5-13.9)	0.286
CRP (mg/l)	13.78 ± 20.07 (1-5.3-72.3)	11.42 ± 13.73 (0.6-5.7-59.7)	12.53 ± 16.87 (0.6-5.6-72.3)	0.858
IL-6 (pg/ml)	10.22 ± 7.64 (1.8-7.7-27.52)	7.61 ± 5.31 (1.7-5.2-19.5)	8.84 ± 6.57 (1.7-7-27.5)	0.299
Serum albumin (g/dl)	3.48 ± 0.43 (2.5-3.6-4)	3.84 ± 0.50 (2.9-3.8-4.9)	3.67 ± 0.50 (2.5-3.7-4.9)	0.026*
Cholesterol (mmol/l)	6.17 ± 1.20 (4-5.88-9)	4.48 ± 1.19 (1.8-4.5-7.5)	5.27 ± 1.46 (1.8-5.27-9)	0.000*
Pro-ANP (nmol/l) 1–98	25.54 ± 13.08 (5.65-24.34-57.25)	24.28 ± 13.72 (4.55-23.8-67.23)	24.87 ± 13.30 (4.5-23.8-67.2)	0.699
NT-proBNP (nmol/l) 1–76	0.28 ± 0.19 (0.056-0.2-0.82)	0.30 ± 0.37 (0.035-0.15-1.98)	0.29 ± 0.30 (0.035-0.20-1.98)	0.312

Primary diagnosis	PD/HD	*n* = 51 pts
Diabetes mellitus	10 (41.67 %)/16 (59.26 %)	26 (51 %)
Hypertensive nephropathy	8 (33.33 %)/6 (22.22 %)	14 (27.4 %)
Interstitial nephropathy	4 (16.67 %)/2 (7.41 %)	6 (11.8 %)
Chronic glomerular disease	2 (8.33 %)/1 (3.70 %)	3 (5.9 %)
Polycystic kidney disease	0 (0 %)/2 (7.41 %)	2 (3.9 %)

* *p* < 0.05, statistically significant; ** 0.05 < *p* < 0.1

Thirteen patients from the initial cohort of 51 (25.5 %) survived the whole 48-month observation period: 10 HD patients (37 %) and 3 PD patients (12.5 %). Table 2 contains the clinical characteristics of the deceased and surviving patients.

**Table 2:** Clinical characteristics of deceased and surviving patients

Characteristics	Deceased pts (*n* = 38)	Surviving pts (*n* = 13)	Mann–Whitney U test *p* value
Age (years)	77.55 ± 3.78	77.23 ± 3.37	0.862
BMI (kg/m2)	25.96 ± 4.40	27.05 ± 3.87	0.418
Duration of dialysis (months)	19.68 ± 16.76	16.62 ± 11.58	0.837
Systolic BP (mmHg)	127.47 ± 14.91	135.69 ± 13.65	0.102
Diastolic BP (mmHg)	78.29 ± 8.94	78.54 ± 8.33	0.893
Residual diuresis (ml/24 h)	540.00 ± 433.08	780.77 ± 313.27	0.034*
Hb (g/dl)	11.29 ± 1.28	11.75 ± 0.85	0.150
CRP (mg/l)	14.30 ± 18.37	7.36 ± 10.32	0.166
IL-6 (pg/ml)	9.72 ± 6.79	6.28 ± 5.34	0.054**
Serum albumin (g/dl)	3.60 ± 0.51	3.88 ± 0.39	0.096**
Cholesterol (mmol/l)	5.38 ± 1.49	4.96 ± 1.38	0.496
Pro-ANP (nmol/l) 1–98	26.52 ± 13.71	20.05 ± 11.16	0.136
NT-proBNP (nmol/l) 1–76	0.31 ± 0.33	0.24 ± 0.17	0.503

* *p* < 0.05, statistically significant; ** 0.05 < *p* < 0.1

In this univariate comparison, the elderly survivors exhibited significantly higher residual diuresis (*p* = 0.034) and slightly lower IL-6 (*p* = 0.054).

The mean annual mortality rate was 28.2 % (37.4 % on PD vs. 20.9 % on HD).

**Fig. 1:**
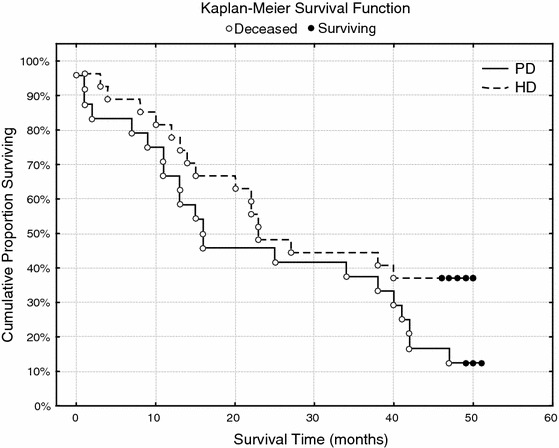
Kaplan–Meier survival function. The difference in mortality between the elderly patients during 4-year observation period according to modality of dialysis treatment—PD versus HD

After the end of the 4-year observation period, the survival of elderly patients was significantly better in the HD-treated group (*p* = 0.045 in Pearson’s χ^2^ test of independence and *p* = 0.044 in Fisher’s exact test; Fig. 1). Type of dialysis modality appeared to be a factor significantly influencing patients’ survival with PD being inferior to HD (*p* = 0.049; Cox F-test). The most common causes of death were as follows: cardiovascular complications 23 pts (60 %) (12 PD/11 HD), infection 12 pts (32 %) (7 PD/5 HD), malignancy 2 pts (10 %) (1 PD/1 HD), and unknown reasons 1 PD pt (3 %).

The independent variables with a significant negative impact on 4-year survival of the followed elderly dialysis patients in the Cox proportional hazard regression model (Table 3) were as follows: higher plasma pro-ANP (*p* = 0.006), lower residual diuresis (*p* = 0.048), and lower systolic BP value (*p* = 0.039).

**Table 3:** Cox proportional hazard regression model

Effect	Estimate	Wald statistics	*p* value
Pro-ANP	0.032873	7.309433	0.006863
Residual diuresis	−0.000995	3.893089	0.048494
Systolic BP	−0.024529	4.226666	0.039802

Dependent variable: survival time from beginning of investigation (*n* = 51)

Referring to the dialysis modality, the Cox proportional hazard regression model showed a significant association of mortality with higher plasma pro-ANP (*p* = 0.008) and lower systolic BP value (*p* = 0.043) in PD patients versus less residual diuresis (*p* = 0.005) and lower BMI (*p* = 0.017) in HD patients. It is worth emphasizing that in this geriatric cohort with age ranging from 71 up to 86 years, the presence of diabetes did not exert a negative impact on survival.

## Discussion

An important element of the study is its performance in the cohort of elderly dialysis patients who survived the first adaptive and disruptive phase of dialysis treatment, having been on maintenance dialysis at least 2 months, with a median period of dialysis therapy before inclusion of 16 months. Other noteworthy distinctive features of the investigated patients were the absence of overt heart failure and symptomatic coronary disease, native arteriovenous fistula as vascular access in all subjects, and nearly equal distribution of individuals between HD and PD treatment. However, two metabolic differences appeared between patients treated by the different dialysis modalities. PD patients exhibited significantly lower albumin and significantly higher cholesterol values. In other aspects, the groups were well matched.

About 25.5 % of the study patients survived the whole 4-year observation period. Ten survivors were on maintenance dialysis and 3 remained on PD. In univariate analysis, the elderly survivors displayed significantly higher residual diuresis and slightly lower IL-6. In multivariate evaluation (Table 3), the independent variables with a negative prognostic impact on 4-year survival were found to be higher plasma pro-ANP, lower residual diuresis, and lower systolic BP value. This reflects the particular importance of meticulous extracellular volume control in the frail elderly population. The consequence of volume overload is left ventricular hypertrophy, and finally, the diminishment of cardiac performance manifested by lower systolic BP [10].

In our study, pro-ANP determination showed a prognostic advantage over NT-proBNP measurement. Elevated pro-ANP level appeared to be an independent predictor of mortality, whereas the NT-proBNP concentration did not exhibit a significant effect. This difference could be caused by diverse mechanisms of pro-ANP and NT-proBNP synthesis. ANP is secreted mainly by the right atrium, while BNP is produced by cardiac ventricles. ANP has been found to be more sensitive to changes in intravascular volume, but BNP level is more related to left ventricular mass and function [11–13]. The prognostic superiority of pro-ANP measurement over NT-proBNP determination in our study population—contradictory to what is observed in cardiac failure patients [14]—is probably the consequence of considering overt cardiac failure as an the exclusion criterion. In the study group created in this manner, homogeneous in terms of cardiac structure, the overhydration reflected by pro-ANP levels appeared to be the decisive factor for survival. We are conscious of our study’s limitation based on the single measurement, and we would emphasize the need for more extensive research before the obtained results could be applied to the general dialysis population.

The mean annual rate of mortality in the study group was 28.2 %.

These high mortality rates mirror the poor clinical situation of elderly dialysis patients. In the recently published dialysis outcomes and practice pattern study (DOPPS), encompassing the largest cohort of hemodialysis patients ≥75 years, the annual mortality rate was 21.4 % [15]. This indicates that our study group, although limited in size, is representative.

After the end of the 4-year observation period, the survival of elderly patients was significantly better in the HD-treated group (Fig. 1). This is in contrast to a recently published Spanish study where no significant differences in survival was found between PD and HD patients [16]. However, it should be underscored that PD patients in our study had a significantly lower albumin level and significantly higher cholesterol concentration, which could negatively affect their survival.

In relation to the dialysis modality, the Cox proportional hazard regression model exhibited a significant association of mortality with higher plasma pro-ANP and lower systolic BP value in PD patients versus less residual diuresis and lower BMI in HD patients. This separate analysis again proves the significance of appropriate volume control in the elderly population, illustrating in the PD group the aforementioned relationship between volume overload and impaired cardiac performance.

Of particular note are the data showing that in HD patients, the drop of residual diuresis was the strongest mortality risk factor. It is according to our knowledge the first such observation in the literature on HD in elderly patients. The issue of residual renal function is widely recognized in PD treatment evaluation, whereas information on the impact of residual renal function on the survival of HD patients is very scanty. We found only one paper showing in the whole HD population, without particular reference to the elderly, that the presence of residual renal function was protective as an independent factor against mortality [17].

Another study brought evidence on the positive relationship between the presence of residual renal function and overall nutritional status in chronic hemodialysis patients [18].

Collectively, these data indicate that residual renal function, which is frequently ignored in HD patients, should be scrupulously evaluated in the elderly, being considered as guidance for dialysis regimen prescription. The value of nourishment for the survival of elderly HD patients was shown in our investigation by revealing lower BMI as an independent mortality risk factor.

The most common causes of death were cardiovascular complications in 60 % of pts, infection in 32 %, and malignancy in 10 %. This breakdown of causes of death is similar to that observed in the largest published cohort of elderly patients in the DOPPS study [15].

## Conclusions

1. Pivotal for the survival of elderly dialysis patients is obtaining the appropriate extracellular volume control.
2. Residual renal function plays a protective role for the survival of elderly HD patients.
